# Comment on 'Lack of evidence for associative learning in pea plants'

**DOI:** 10.7554/eLife.61141

**Published:** 2020-09-10

**Authors:** Monica Gagliano, Vladyslav V Vyazovskiy, Alexander A Borbély, Martial Depczynski, Ben Radford

**Affiliations:** 1 The Biological Intelligence (BI) Lab, School of Life and Environmental Sciences, University of SydneyCamperdownAustralia; 2 School of Science and Engineering and School of Creative Industries, University of the Sunshine CoastMaroochydoreAustralia; 3 Department of Physiology, Anatomy and Genetics, University of OxfordOxfordUnited Kingdom; 4 Institute of Pharmacology and Toxicology, University of ZurichZurichSwitzerland; 5 Australian Institute of Marine Science, The Oceans Institute, University of Western AustraliaCrawleyAustralia; Johns Hopkins UniversityUnited States; University of LausanneSwitzerland

**Keywords:** associative learning, memory, plant growth, pisum sativum, replication, phototropism, Other

## Abstract

In 2016 we reported evidence for associative learning in plants (Gagliano et al., 2016). In view of the far-reaching implications of this finding we welcome the attempt made by Markel to replicate our study (Markel, 2020). However, as we discuss here, the protocol employed by Markel was unsuitable for testing for associative learning.

## Introduction

Testing for associative learning relies on the pairing of an unconditioned stimulus (US) with a conditioned stimulus. To be effective, the stimulus used as an US must invariably elicit a response (in Pavlov's classical experiment in dogs the presentation of food elicited invariably salivation). In our study (Gagliano et al., 2016) we used blue light as the US which caused consistently a growth of the plant in the direction of the last presentation of the light (100% phototropic response). This was not the case in the study by Markel, where only a slight bias towards the last presentation of light was obtained (Markel, 2020). Since light was not an effective US in the study by Markel, it is not surprising that no distinct associative learning was observed.

In our study we also encountered conditions in which light was not an effective US. Thus, in the second series of our experiments, we tested the response of the plants in different circadian phases (light, light-dark, dark: see Figure 3 of Gagliano et al., 2016). Whereas the 100% phototropic response was obtained in the light phase, it was attenuated or abolished in the other two protocols. Consequently, no associative learning could be shown in those conditions.

We offer the following potential explanation for the lack of a consistent phototropism in Markel, 2020. Our study was conducted inside a completely dark 5.3 m^2^ room, where individual Y-mazes were positioned at ample distance (~20 cm radius) from each other. This was necessary to ensure that a plant inside its maze could only receive the blue light we directionally delivered within each maze at set specific times, and was completely shielded from light sources elsewhere. The lack of darkness in the study by Markel (see Figure 1—figure supplement 1C in Markel, 2020) is a major departure from our original design. We surmise that by inadvertently allowing individual plants to be exposed to light arriving from multiple sources within a 1.5 m^2^ growth cabinet (e.g. light leaking from mazes positioned too close to each other or reflecting from the chamber’s walls), the set up used by Markel could have resulted in random growth patterns, unrelated to the behaviour the experimental treatments were designed to test for, thereby confounding the results.

## Data Availability

No data was generated for this study.
